# Effects of patient age and choice of antisecretory agent on success of eradication therapy for *Helicobacter pylori* infection

**DOI:** 10.3164/jcbn.16-86

**Published:** 2017-02-16

**Authors:** Toshihiro Nishizawa, Hidekazu Suzuki, Ai Fujimoto, Hiroto Kinoshita, Shuntaro Yoshida, Yoshihiro Isomura, Akira Toyoshima, Takanori Kanai, Naohisa Yahagi, Osamu Toyoshima

**Affiliations:** 1 Gastroenterology, Toyoshima Endoscopy Clinic, Tokyo 157-0066, Japan; 2 Division of Research and Development for Minimally Invasive Treatment, Cancer Center, Keio University School of Medicine, 35 Shinanomachi, Shinjuku, Tokyo 160-8582, Japan; 3 Division of Gastroenterology and Hepatology, Department of Internal Medicine, Keio University School of Medicine, 35 Shinanomachi, Shinjuku, Tokyo 160-8582, Japan; 4 Medical Education Center, Keio University School of Medicine, 35 Shinanomachi, Shinjuku, Tokyo 160-8582, Japan; 5 Department of Gastroenterology, Graduate School of Medicine, The University of Tokyo, Tokyo 113-0033, Japan; 6 Department of Gastroenterology, Kanto Central Hospital, Tokyo 158-8531, Japan; 7 Department of Surgery, Japanse Red Cross Medical Center, Tokyo 150-8935, Japan

**Keywords:** *H. pylori*, eradication, age, vonoprazan, proton pump inhibitor

## Abstract

The effects of patient age on the efficacy of eradication treatment for *Helicobacter pylori* (*H. pylori*) remain unclear. The present study aimed to determine whether age affects eradication therapy involving vonoprazan, a novel potassium-competitive acid blocker (PCAB). We reviewed the cases of 3,261 patients who were administered first-line and second-line *H. pylori* eradication therapy at Toyoshima Endoscopy Clinic. The first-line treatment was clarithromycin and amoxicillin combined with a proton pump inhibitor (PPI) or a PCAB. The second-line treatment was metronidazole and amoxicillin combined with a PPI or PCAB. The patients were divided into a young to middle-aged group (age ≤50 years) and an older group (age >50 years) as well as into PPI and PCAB groups. The PPI-clarithromycin-amoxicillin regimen demonstrated a significantly lower *H. pylori* eradication rate than the PCAB-clarithromycin-amoxicillin regimen (*p*<0.001). With the PPI-clarithromycin-amoxicillin regimen, the eradication rate in the young to middle-aged group was significantly lower than that in the older group (*p*<0.001). Lastly, age had no impact on the eradication rate of PCAB-based therapy or metronidazole-based therapy. In conclusion, with clarithromycin-based triple therapy, PCAB is a better choice of antisecretory agent compared to PPIs, especially in young to middle-aged patients.

## Introduction

Eradication of *Helicobacter pylori* (*H. pylori*) infection has been reported as an effective strategy in the treatment of peptic ulcers and gastric mucosa-associated lymphoid tissue lymphoma as well as in the prevention of gastric cancer.^(1–6)^ The first-line eradication regimen of clarithromycin combined with amoxicillin and a proton pump inhibitor (PPI) is covered under national health insurance in Japan.^(7)^ However, the success rate of this regimen has recently dropped to ≤75% because of an increasing incidence of clarithromycin resistance.^(8–10)^ The second-line regimen, using metronidazole combined with amoxicillin and a PPI, is also covered under national health insurance in Japan,^(11)^ and the prevalence of *H. pylori* resistance to metronidazole in Japan is 5–12%, while the success rate of this second-line regimen has remained constant at approximately 90%.^(12,13)^

In 2015, vonoprazan, a member of a new class of potassium-competitive acid blockers (PCABs), was released and approved for use in *H. pylori* eradication in Japan. Murakami *et al.*^(14)^ have reported the superiority of vonoprazan over lansoprazole for first-line *H. pylori* eradication therapy, with an eradication rate of 93% for vonoprazan and 76% with lansoprazole (*p*<0.01). Mamori *et al.*^(15)^ reported that first-line eradication failures occurred more frequently in patients aged less than 50 years than in those aged over 50. However, it remains unknown whether patient age affects PCAB-based therapy and metronidazole-based treatment. The aim of the present study was to determine whether age affects the eradication of *H. pylori* in routine clinical practice.

## Methods

### Subjects

We retrospectively reviewed the data of 3,261 patients who were administered first- and second-line *H. pylori* eradication therapy at the Toyoshima Endoscopy Clinic between February 2002 and June 2016. *H. pylori* positivity in these patients was confirmed from the results of the ^13^C-urea breath test, stool antigen test, or the presence of *H. pylori*-specific IgG antibodies in the serum.^(16)^

First-line triple therapy included 200 mg clarithromycin, 750 mg amoxicillin, and an antisecretory agent (30 mg lansoprazole, 10 mg rabeprazole, or 20 mg vonoprazan) twice daily for 1 week. Eradication was confirmed using the urea breath test at least 4 weeks after the treatment. The cut-off value for the urea breath test was 2.5%. Patients in whom first-line therapy failed to eradicate the pathogen received second-line triple therapy with 250 mg metronidazole, 750 mg amoxicillin, and an antisecretory agent (30 mg lansoprazole, 10 mg rabeprazole, or 20 mg vonoprazan) twice daily for 1 week. The antisecretory agent was chosen by the physician in charge. Favorable eradication data for vonoprazan were released in February 2015, and this has been the antisecretory agent of choice since March 2015.

The success rates of eradication were assessed using intention-to-treat (ITT) and per protocol (PP) analyses. Patients who did not return to the clinic to receive a urea breath test for evaluating the results of eradication therapy were excluded from the PP analysis.

### Statistical analysis

Patients were classified into a young to middle-aged group (age ≤50 years) and an older group (age >50 years) to examine the effect of age on treatment success. Further, patients were also classified into the PPI (lansoprazole or rabeprazole) and PCAB group (vonoprazan) to examine the effect of antisecretory agent on treatment success.

Differences between the groups were compared using the chi-squared test for categorical variables. A *p* value of less than 0.05 was considered statistically significant. Data were analyzed using the Stat Mate IV software (ATOMS, Tokyo, Japan).

### Ethics

The study was approved by an external ethics committee, and informed consent was obtained from all patients. The University Hospital Medical Information Network clinical trial registration number is UMIN000018541.

## Results

Table 1 shows the demographic data of the 3,261 patients included in the present study, while Table 2 shows the success rate of *H. pylori* eradication therapy. The eradication rate of the first-line clarithromycin-based triple therapy with PPIs was significantly lower than that with PCAB (*p*<0.001, in ITT and PP analyses). However, the rate of the second-line metronidazole-based triple therapy did not differ significantly between the PPI and PCAB groups.

The eradication rate of the first-line therapy with PPIs was significantly lower for the young to middle-aged group than for the elder group (*p*<0.01 for ITT and *p*<0.001 for PP). In contrast, for the first-line therapy with PCAB and the second-line metronidazole-based triple therapy, the eradication rates were similar in both age groups.

**Fig. 1:**
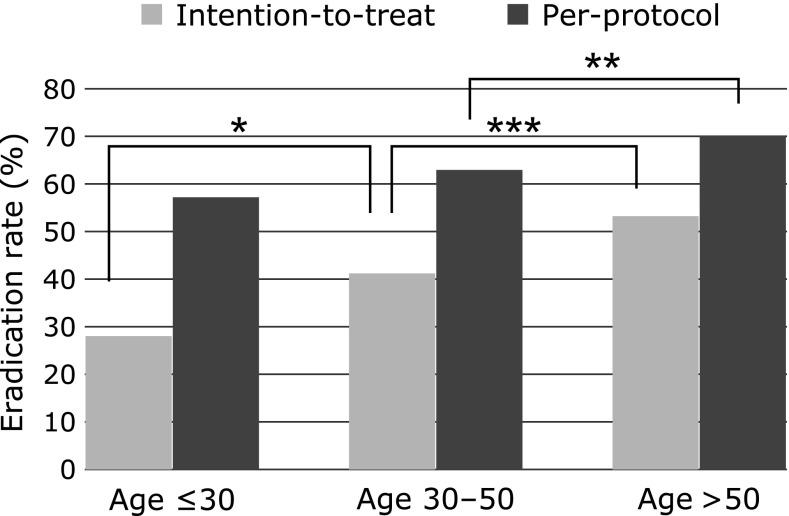
The eradication rates of clarithromycin-based triple therapy with PPIs in young (≤30 years), middle-aged (30–50 years), and old patients (>50 years). *p<0.05, **p<0.01, ***p<0.001.

The eradication rates of clarithromycin-based triple therapy with PPIs were increased stepwise in young (≤30 years), middle-aged (30–50 years), and old patients (>50 years) (Fig. 1).

The data of side effects were available in 917 patients. The incidence of side effects was 5.7% (30/529) in patients receiving the first-line clarithromycin-based triple therapy with PPIs, and 8.4% (13/154) in patients receiving the first-line clarithromycin-based triple therapy with PCAB. The incidence of side effects was 3.3% (6/184) in patients receiving the second-line metronidazole-based triple therapy with PPIs, and 6% (3/50) in patients receiving the second-line metronidazole-based triple therapy with PCAB. For the incidence of side effects, there was no significant difference between PPIs and PCAB. In these cases, the side effects were mild and mainly included diarrhea, skin rash, or stomatitis.

## Discussion

The results of the present study showed that first-line clarithromycin-based triple therapy with PPIs demonstrated a significantly lower eradication rate than the same therapy with PCAB. Further, clarithromycin-based triple therapy with PPIs failed significantly more frequently in young to middle-aged patients (age ≤50 years) than in older patients (age <50 years). However, age had no impact on the eradication rate for PCAB-based therapy or metronidazole-based therapy.

In their 2008–2009 study, Okamura *et al.*^(17)^ reported that the rates of clarithromycin resistance were 51.6%, 46.8%, and 25.5% in young (≤30 years), middle-aged (30–50 years), and old patients (>50 years). As indicated by these values, the rate of resistance was significantly higher in the young (*p* = 0.03) and middle-aged (*p*<0.01) patients than the old patients. However, metronidazole resistance was similar among the three groups.

We believe that the higher rate of clarithromycin resistance in young to middle-aged group adversely affected the success rate of clarithromycin-based triple therapy with PPIs. However, surprisingly, this higher resistance rate did not affect the success rate of clarithromycin-based triple therapy with PCAB.

Murakami *et al.*^(14)^ reported that the eradication rate of first-line clarithromycin-based triple therapy was significantly higher with vonoprazan than with lansoprazole in patients infected with clarithromycin-resistant strains of *H. pylori* (82.0% vs 40.0%; *p*<0.0001). Vonoprazan may compensate for clarithromycin resistance, but the underlying mechanism remains unclear. One explanation could be synergistic actions between vonoprazan and the antimicrobials used, in that *H. pylori* is more susceptible to antimicrobials when its replicative capability is restored at pH values exceeding 6, which are the levels facilitated by vonoprazan.^(18)^

The present study has some limitations. First, many patients did not return to the clinic to undergo a urea breath test for evaluating the results of eradication therapy because of routine clinical practice. Yogeswaran *et al.*^(19)^ also reported that confirmation of eradication was lacking in a significant proportion of patients in clinical practice. Second, this study was retrospective, and adverse events were partially available. A prospective follow-up study should be performed to confirm our results.

In conclusion, the PPI-clarithromycin-amoxicillin regimen showed a significantly lower *H. pylori* eradication rate than the PCAB-clarithromycin-amoxicillin regimen did. Additionally, with the former, the eradication rate in the young to middle-aged group was significantly lower than that in the older group of patients. Thus, with clarithromycin-based triple therapy, PCAB is a better choice of antisecretory agent than PPIs, especially in young to middle-aged patients (age ≤50 years).

## Figures and Tables

**Table 1:** Demographic data of patients undergoing eradication therapy for *H. pylori*

	Regimen	Number	Age	Sex (M/F)
Total	Triple therapy	3,261	52.3 ± 7.5	1,508/1,753

First-line treatment	Lansoprazole or rabeprazole	2,173	52.7 ± 13.8	1,012/1,161
	Clarithromycin, amoxicillin			
	
	Vonoprazan	353	50.4 ± 13.3	138/215
	Clarithromycin, amoxicillin			

Second-line treatment	Lansoprazole or rabeprazole	650	51.7 ± 13.0	322/328
	Metronidazole, amoxicillin			
	
	Vonoprazan	85	53.5 ± 13.3	36/49
	Metronidazole, amoxicillin			

M, male; F, female.

**Table 2:** Success rate of *H. pylori* eradication therapy

	Regimen	Age	Per protocol	Intention to treat
First-line treatment	Lansoplazole or rabeprazole	All	66.8% (1,024/1,532)	47.1% (1,024/2,173)
	Clarithromycin	≤50	62.6% (403/644)	40.1% (403/1,006)
	Amoxicillin	>50	69.9% (621/888)^#^	53.2% (621/1,167)^##^
	
	Vonoprazan	All	89.4% (220/246)**	62.3% (220/353)**
	Clarithromycin	≤50	89.4% (118/132)**	60.5% (118/195)**
	Amoxicillin	>50	89.5% (102/114)**	64.5% (102/158)*

Second-line treatment	Lansoprazole or rabeprazole	All	90.5% (479/529)	73.7% (479/650)
	Metronidazole	≤50	90.8% (216/238)	68.4% (216/316)
	Amoxicillin	>50	90.4% (263/291)	78.7% (263/334)
	
	Vonoprazan	All	96.8% (61/63)	71.8% (61/85)
	Metronidazole	≤50	96.8% (30/31)	78.9% (30/38)
	Amoxicillin	>50	96.9% (31/32)	66.0% (31/47)

^#^*p*<0.01, ^##^*p*<0.001, Age >50 group vs age ≤50 group. *p<0.01, **p<0.001, Vonoprazan based regimen vs lansoplazole or rabeprazole based regimen.
